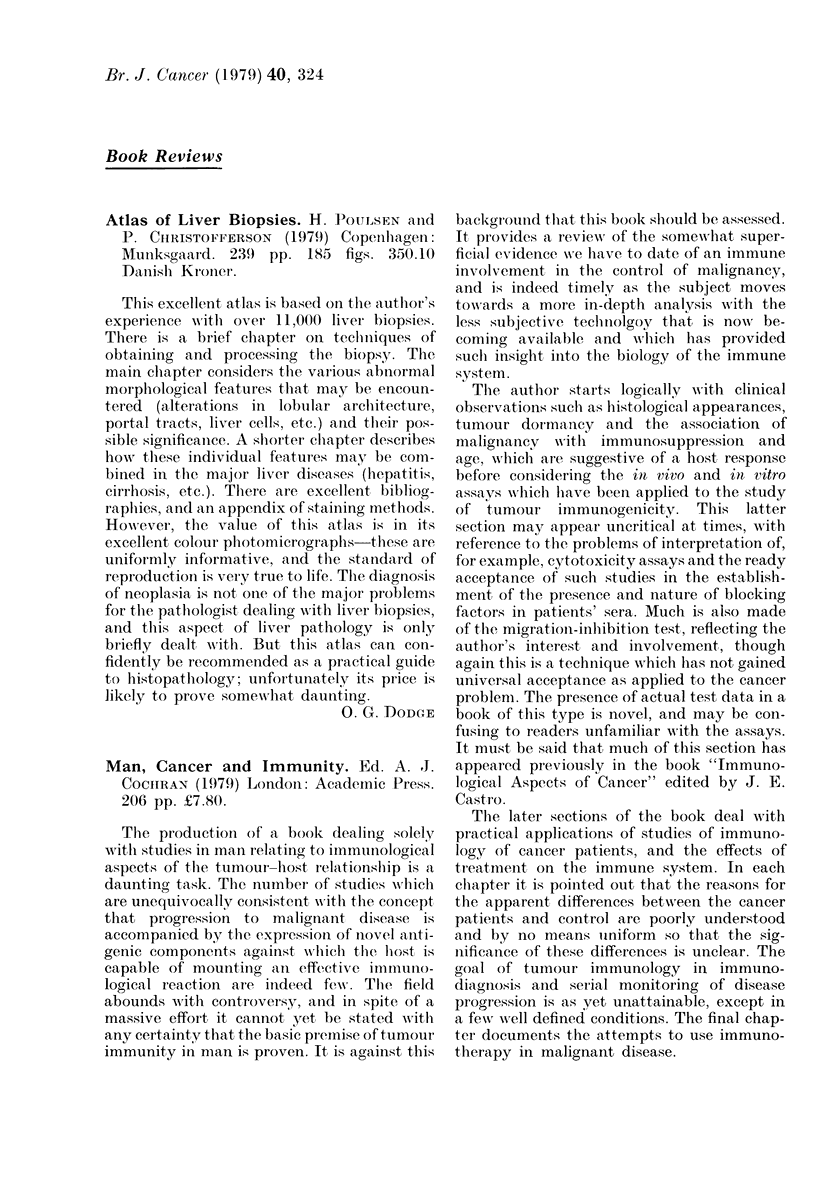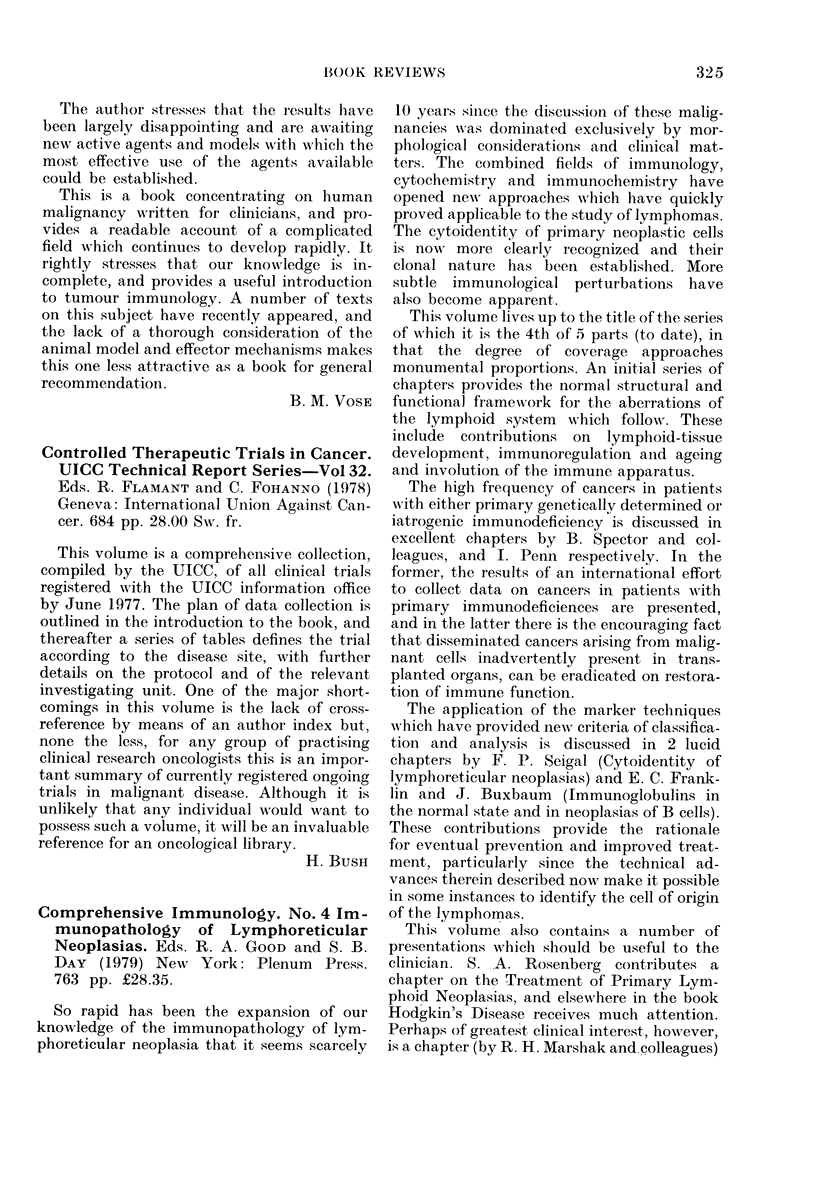# Man, Cancer and Immunity

**Published:** 1979-08

**Authors:** B. M. Vose


**Man, Cancer and Immunity.** Ed. A. J. COCHRAN (1979) London: Academic Press. 206 pp. £7.80.

The production of a book dealing solely with studies in man relating to immunological aspects of the tumour–host relationship is a daunting task. The number of studies which are unequivocally consistent with the concept that progression to malignant disease is accompanied by the expression of novel antigenic components against which the host is capable of mounting an effective immunological reaction are indeed few. The field abounds with controversy, and in spite of a massive effort it cannot yet be stated with any certainty that the basic premise of tumour immunity in man is proven. It is against this background that this book should be assessed. It provides a review of the somewhat superficial evidence we have to date of an immune involvement in the control of malignancy, and is indeed timely as the subject moves towards a more in-depth analysis with the less subjective technolgoy that is now becoming available and which has provided such insight into the biology of the immune system.

The author starts logically with clinical observations such as histological appearances, tumour dormancy and the association of malignancy with immunosuppression and age, which are suggestive of a host response before considering the *in vivo* and *in vitro* assays which have been applied to the study of tumour immunogenicity. This latter section may appear uncritical at times, with reference to the problems of interpretation of, for example, cytotoxicity assays and the ready acceptance of such studies in the establishment of the presence and nature of blocking factors in patients' sera. Much is also made of the migration-inhibition test, reflecting the author's interest and involvement, though again this is a technique which has not gained universal acceptance as applied to the cancer problem. The presence of actual test data in a book of this type is novel, and may be confusing to readers unfamiliar with the assays. It must be said that much of this section has appeared previously in the book "Immunological Aspects of Cancer" edited by J. E. Castro.

The later sections of the book deal with practical applications of studies of immunology of cancer patients, and the effects of treatment on the immune system. In each chapter it is pointed out that the reasons for the apparent differences between the cancer patients and control are poorly understood and by no means uniform so that the significance of these differences is unclear. The goal of tumour immunology in immunodiagnosis and serial monitoring of disease progression is as yet unattainable, except in a few well defined conditions. The final chapter documents the attempts to use immunotherapy in malignant disease.

The author stresses that the results have been largely disappointing and are awaiting new active agents and models with which the most effective use of the agents available could be established.

This is a book concentrating on human malignancy written for clinicians, and provides a readable account of a complicated field which continues to develop rapidly. It rightly stresses that our knowledge is incomplete, and provides a useful introduction to tumour immunology. A number of texts on this subject have recently appeared, and the lack of a thorough consideration of the animal model and effector mechanisms makes this one less attractive as a book for general recommendation.

B. M. VOSE